# Reproductive performance in houbara bustard is affected by the combined effects of age, inbreeding and number of generations in captivity

**DOI:** 10.1038/s41598-021-87436-z

**Published:** 2021-04-09

**Authors:** Robin Rabier, Loïc Lesobre, Alexandre Robert

**Affiliations:** 1 Reneco International Wildlife Consultant LLC, Abu Dhabi, United Arab Emirates; 2 Centre d’Ecologie et des Sciences de la Conservation (CESCO), Muséum national d’Histoire naturelle, Centre National de la Recherche Scientifique, Sorbonne Université, 57 rue Cuvier, CP 135, 75005 Paris, France; 3 Emirates Center for Wildlife Propagation, Missour, Morocco

**Keywords:** Conservation biology, Genetics, Inbreeding, Senescence

## Abstract

Although captive breeding programs are valuable for conservation, they have been shown to be associated with genetic changes, such as adaptation to captivity or inbreeding. In addition, reproductive performance is strongly age-dependent in most animal species. These mechanisms that potentially impact reproduction have often been studied separately, while their interactions have rarely been addressed. In this study, using a large dataset of nine male and female reproductive parameters measured for 12,295 captive houbara bustards (*Chlamydotis undulata undulata*) over 24 years, we investigated the relative and interactive effects of age, inbreeding and number of generations in captivity on reproduction. We clearly identified (1) senescence patterns in all parameters studied; (2) negative effects of inbreeding on sperm characteristics, display behavior, egg weight, egg volume and hatching probability; and (3) changes in phenotypic values for seven parameters according to number of generations in captivity. However, the effect sizes associated with age were substantially greater than those associated with inbreeding and number of generations in captivity. Beyond the independent effects of these three factors on reproductive parameters, the results highlighted their interactive effects and thus the importance of integrating them in the design of genetic management plans for conservation breeding programs.

## Introduction

As complementary tools to in situ conservation measures, conservation breeding programs are now recognized as essential actions for the conservation of species and populations^1^. These programs must achieve demographic goals (e.g., production of individuals to supplement free-ranging populations) as well as genetic goals (e.g., maintenance of the initial genetic diversity) through appropriate genetic management^2–4^. Indeed, captive conditions (e.g., limited carrying capacity and controlled environment) increase the potential for genetic changes associated with genetic drift^5^, inbreeding^6^ and adaptation to captivity^7^. Inbreeding, or mating among relatives, is often associated with genetic deterioration^8^, which can lead to an altered fitness (inbreeding depression) that can in turn jeopardize the success of supplementation programs based on captive breeding^6,9^. Inbreeding depression can be caused by deleterious recessive or partially recessive alleles (dominance theory) and/or to an increase in homozygosity at loci coding for fitness-related traits, with an advantage observed for heterozygotes (overdominance theory)^10^. Adaptation to captivity occurs through natural selection and is an evolutionary response to changes in selection pressure due to captive conditions^7^. Such changes may be related to advantage conferred by certain phenotypes and behaviors in captivity, such as tamed individuals or the best breeders (adaptation to captivity sensu stricto). In addition, a relaxation of selection pressure linked to the controlled conditions of the captive environment (e.g., absence of predators, veterinary care and ad libitum feeding) can occur, which may result in an increase in phenotype variance and phenotypes that would be detrimental in the natural environment^11^. Adaptation to captivity is influenced by population size^7^, selection intensity^12^, population genetic variability^7^, gene flow between wild and captive populations^13^ and number of generations in captivity, which determines the extent of evolutionary changes^14^. Previous studies have highlighted that under extreme conditions (e.g., absence of genetic management of the captive population and strong selective pressure), changes in fitness components arising from adaptation to captivity can be phenotypically apparent after only one or a few generations in captivity^15,16^. However, changes in phenotypic means are not necessarily related to an adaptive genetic response to selection and might be the result of phenotypic plasticity^17,18^.

In most animal species, reproductive performance (i.e., survival and reproduction) increases at an early age in relation to growth, physiological maturation and learning processes^19^ before declining at older ages in relation to senescence, which corresponds to an age-dependent physiological deterioration of organism^20,21^. As a result, a pattern of increased performance at young ages and then progressively decreased performance is expected for a wide variety of traits^6,22,23^. According to senescence theories, the progressive decline in performance with age can be caused by an accumulation of deleterious mutations (mutation accumulation theory) and/or an accumulation of alleles that are beneficial at young ages but have deleterious effects at old ages (antagonistic pleiotropy theory)^21^ and/or a trade-off among growth, reproduction and DNA repair maintenance (disposable soma theory)^21,24^. Since senescence increases individual sensitivity to intrinsic and extrinsic factors^20^, it has the potential for significant interaction with inbreeding and adaptation to captivity^25,26^. Indeed, some theories describing senescence are based on the assumption that natural selection against deleterious alleles decreases with age^27^, thus, it is expected that changes in natural selection in captivity can lead to an alteration in individual senescence trajectories. Simultaneously, both the mutation accumulation theory of aging and the dominance theory of inbreeding depression predict an accumulation of deleterious or semi deleterious alleles and are thus expected to interact^28–30^.

In this context, we aimed to quantify and compare the effects of age, inbreeding, number of generations in captivity (as a measure of potential evolutionary response to captive conditions) and their interactions on the reproductive performance of birds. The study model was a large captive population of North African houbara bustard *Chlamydotis undulata undulata* (Jacquin 1784, hereafter houbara) that have been maintained in captivity and managed to maximize the maintenance of genetic diversity for 24 years^31,32^. Our study is at the interface of previous research on houbara that have highlighted (1) a negative effect of inbreeding on behavioral phenotypes^33,34^, hatching success, post hatching mortality and growth^35^; (2) the senescence of reproductive parameters in the free-ranging population^36^ but also in the captive population^37–40^; (3) the efficient maintenance of genetic diversity and minimization of inbreeding within the captive population^32^; and (4) intergenerational variations in reproductive parameters^41,42^. Within this framework, we were particularly interested in (1) quantifying the relative effects of age, inbreeding and number of generations in captivity on reproductive performance and (2) assessing their potential interactions. During this study, a set of reproductive parameters, which was different for each sex, was measured in 7242 females and 5053 males, with an individual number of generations in captivity ranging from 0 (founders) to 5.1. Data were measured between 1997 and 2019. For males, we used the mass motility index, the number of sperm per ejaculate and the number of displaying days. The number of displaying days corresponded to the number of days where courtship display behavior was recorded for a male. This parameter was used because it is a crucial behavior in a lekking species with strong sexual selection and female mate choice, such as the houbara^43,44^. For females, we used the number of eggs laid, egg weight, egg volume, hatching probability and hatching weight, which are related to females’ reproductive success. In addition, we analyzed egg elongation, which has been shown to vary with environmental conditions^45^ and therefore affects hatching success^46,47^. According to our knowledge of the species and the general theoretical and empirical knowledge mentioned above, the following predictions were formulated:

1. A negative relationship exists between inbreeding and reproductive parameters^33,34^.
2. Although genetic management aims to minimize artificial selection in captivity, the number of offspring eventually produced by each captive breeder is necessarily affected by its individual breeding potential within the captive environment, e.g., the quality and quantity of sperm and the number of eggs laid^41,42^, which might result in the selection of these parameters. Thus, we predicted (1) an increase in phenotypic values of male parameters (i.e., number of sperm, mass motility index and the number of displaying days) with the number of generations in captivity and (2) an increase in the number of eggs laid with the number of generations in captivity. We did not formulate any directional predictions for other parameters (i.e., egg weight, egg volume, egg elongation, hatching probability and hatching weight).
3. The occurrence of a senescent pattern with variation in phenotypic values^37–40^.
4. A positive interaction between inbreeding and age accelerates senescence in most inbred individuals^28–30^.

## Methods

### Studied species and population

Houbara is a threatened bird originating from a region covering North Mauritania to Egypt. Because of human activities, the species suffered a strong decline in population size in the 1990s^48^, which led to its classification as “vulnerable” on the Red List of the International Union for Conservation of Nature^49^. Houbara is also listed under Appendix 1 of the Convention of the International Trade of Endangered Species (CITES, https://cites.org/eng/node/20646). Therefore, the International Fund for Houbara Conservation (IFHC, www.houbarafund.org) created a conservation breeding program in 1996 in Missour (Morocco), namely, the Emirates Center for Wildlife Propagation (ECWP, www.ecwp.org). The goal of the ECWP is to restore a viable free-ranging population of houbara^50^ by combining in situ (e.g., ecological studies and hunting regulation) and ex situ conservation measures (e.g., preservation of the species gene pool and provision of birds for translocations). The ECWP’s captive population of houbara was established with founders collected in Algeria in 1986 and 1987, and their descendants, transferred from the National Wildlife Research Center (Taïf, Saudi Arabia). Further founders resulting from wild egg collections in Eastern Morocco were added from 1996 to 1997 (N = 115 eggs), 2002 to 2009 (N = 479 eggs) and 2015 to 2017 (N = 191 eggs). The captive population increased from 296 individuals in 1996 to 7768 in 2019. Between 1997 and 2017, 198,556 chicks hatched in captivity, of which 133,423 were released into the wild and the others were kept in captivity to renew the breeding flock. The captive population of houbara is maintained in a captive-free-ranging system with regular exchanges between captive and free-ranging populations through supplementation releases of captive-bred birds and regular collections of founders from the wild to prevent genetic drift^5^ and minimize risks of adaptation to captivity^51^. The captive population of ECWP is divided into three geographically distant sites. However, genetic management is applied to the whole population. Within the ECWP, reproduction is performed artificially, including sperm collection, insemination and incubation^52^. Such work along with the individual housing of birds allowed us to build complete pedigrees (98.8% complete on average). Pairing management follows strict genetic management based on the pedigree analyses to (1) minimize the mean kinship within the captive population^2^, (2) avoid inbreeding and (3) equalize family size^53^.

### Ethics statement

Breeding and experimental protocols were approved by Moroccan authorities (Ministère de l’Agriculture, Développement Rural et des Pêches Maritimes, Direction Provinciale de l’Agriculture de Boulemane, et Service Vétérinaire; Nu DPA/48/285/SV) under permit number 01-16/VV; OAC/2007/E; Ac/Ou/Rn. Thus, experiments were performed in accordance with relevant guidelines. All data were extracted from an existing database handled by the Emirates Center for Wildlife Propagation. This study complies with the ARRIVE guidelines^54^.

### Reproductive parameters

We analyzed three reproductive parameters of males, i.e., the number of displaying days, the number of sperm per ejaculate and the mass motility index, along with six reproductive parameters of females, i.e., the total number of eggs laid per female during a year, egg weight, egg elongation, egg volume, hatching probability and hatching weight. The parameters of individuals were analyzed yearly for the breeding season between January 1st and June 14th from 2003 to 2019 for males and between January 1st and August 31st from 1997 to 2019 for females. Note that we also included December for the number of displaying days to include early displaying males. During that period, the display behavior of males was recorded daily and the number of displaying days was the total number of days where at least one courtship display behavior was recorded throughout a breeding season. Males were included in the sample from their first breeding year; subsequently, a zero was applied for every year where no courtship display was observed. The mass motility index, a proxy of sperm quality and fertility potential in the species^55^, ranged from 0 (no motile sperm) to 5 (almost 100% of sperm showing rapid movement)^52^. The number of sperm in the ejaculate (in million) was assessed using a colorimeter and a specific calibration to convert optical density to the concentration of spermatozoa^56^. Details of sperm collection and characterization can be found in the Supplementary Methods online. For females, the number of eggs laid was recorded throughout a season, and a zero was applied for every non laying year of a female. Females were included in the sample from their first breeding season. Eggs were collected daily, and their morphology was measured before being placed in artificial incubators for a period of 23 days. Each egg was weighed (in grams) and egg elongation was computed as the ratio between egg length and width. Egg volume was computed as $V=(0.51\times {width}^{2}\times length)/1000$, where 0.51 is the average egg volume coefficient computed from 115 species^57^. Hatching probability was modeled using a binomial distribution function since the initial variable was coded as 0 (unhatched egg) or 1 (hatched egg). Each chick was weighed (in gram) just after hatching.

### Age, inbreeding and number of generations in captivity

Pedigree-based individual inbreeding coefficient F, which is the probability that two homologous genes of an individual are identical by descent^8^, was computed using the package optiSel 2.0.2^58^ in R 3.6.1^59^. Founders of the captive population were considered neither inbred nor related, and their F was set to zero^32^. The number of generations in captivity was computed as the average number of its parents plus one^60^. The number of generations in captivity of founders was set to zero. Individual age, in years, was computed for each record. Descriptive statistics of age, inbreeding and number of generations in captivity are provided in Table 1.

**Table 1:** Descriptive statistics of the three main explanatory variables used to analyze reproductive parameters within the ECWP’s captive population of houbara.

	Age		Inbreeding		Number of generations in captivity	
	Males	Females	Males	Females	Males	Females
Min	1	1	0	0	0	0
Mean (SD)	6.0 (3.7)	6.6 (3.9)	0.004 (0.014)	0.004 (0.015)	2.4 (0.9)	2.4 (1.0)
Max	32	28	0.25	0.25	4.8	5.1

*Min*. minimum, *Max*. maximum, *SD* standard deviation.

### Statistics

All statistics and figures were carried out in R 3.6.1^59^. Reproductive parameters were analyzed through generalized linear mixed-effects models using the package glmmTMB 0.2.3^61^. The mass motility index, egg weight, egg elongation, egg volume and hatching weight were analyzed using a Gaussian distribution function (identity link function), while the number of sperm and the number of displaying days were analyzed using a negative binomial type I function (log link function) since they were count over dispersed data. Hatching probability was analyzed using a binomial function (logit link function). Finally, the number of eggs laid was analyzed using a zero-truncated negative binomial distribution function considering the large amount of 0 (7112 on 39,693 records). First, this model considered the variable to be zero versus nonzero and a binomial model was applied (logit link function). Then the nonzero data were modeled using a negative binomial function (log link function). Note that the analysis of the mass motility index using the Gaussian distribution function was inadequate since the variable was not a continuous quantitative variable, thus implying that predicted values must be considered with caution. An ordinal regression model would be ideal; however, convergence problems were observed when we attempted to fit this type of model. Ordinal values of the mass motility index reflect a certain amount of quantitative variation in the mass motility index. For each parameter, three main explanatory variables were considered quantitative fixed-effect variables: individual age, individual inbreeding and individual number of generations in captivity. Quadratic terms were added for these main response variables to allow for curvilinear relationships. Complex relationships might be expected for age; thus, additional figures based on generalized additive mixed-effects models are presented in the Supplementary Figure S2 online. All first-order interaction terms among these three variables were added. In addition, fixed-effect control covariates were included. Thus, the location was included to control for a potential site effect. In addition, the delay between two eggs for females (Delay_Egg) and between two sperm collections for males (Delay_Sperm) were included to test for the potential effect of time between two egg-laying/sperm-collections (all delays expressed in days). The number of sperm collection attempts per male per year (NbVisits) was also included. The origin wild-bred (individual resulting from egg collection in the wild) or captive-bred of individuals was also included. The serial number of both eggs and ejaculates during the year (N_Egg and N_Sperm, respectively) was included to control for a potential effect of the advancement during the year. Finally, random effect factors were included to control for cohort effects using the birth year and for interannual variations using the year of record. Individual identity was also included as a random effect variable since there were multiple records for each individual. Continuous variables were scaled before fitting the models. Model structures can be found in the Supplementary Table S1. Sample sizes and descriptive statistics for each reproductive parameter are provided in Table 2. Model selection was performed through the drop technique, in which terms are removed one by one, and comparisons of models are based on minimizing the Akaike Information Criteria. This method was applied using the *drop1* function of the package stats 3.6.1^59^. Note that the three main explanatory variables (age, inbreeding and number of generations in captivity) were retained in the final models, even if non-significant. Subsequently, the models were validated by checking the homogeneity and normality of the residuals and multicollinearity of the remaining variables using the package performance 0.3.0^62^. If the effect of a variable was significant, its effect on the reproductive parameters was predicted using the package ggeffects 0.13^63^ or function *predict* of the package stats 3.6.1^59^ for interaction predictions. Graphs presenting predictions were only produced for the significant effects of age, inbreeding and number of generations in captivity. Figures were created using the package ggplot2 3.3.2^64^.

**Table 2:** Descriptive statistics for each reproductive parameter analyzed within the ECWP’s captive population of houbara.

Reproductive parameters	Min	Mean (SD)	Max	Number of data points	Number of individuals
Mass motility index	0	3.5 (0.9)	5	591,924	4168
Nb. of sperm (millions)	0	32.3 (25.2)	652.9	592,405	4170
Nb. of displaying days	0	59.6 (44.8)	292	31,925	4802
Nb. of eggs laid	0	7.1 (5.8)	35	39,693	7236
Hatching probability	0	0.7 (0.4)	1	218,999	5840
Egg weight (g)	20.5	62.4 (6.2)	96.6	85,314	4545
Hatching weight (g)	22	41.1 (4.4)	74.4	134,675	5013
Egg elongation	0.6	1.4 (0.1)	2.3	107,938	5424
Egg volume	9.1	56.8 (5.9)	151.7	107,938	5424

*Min*. minimum, *Max*. maximum, *SD* standard deviation.

## Results

On average, the 5053 males were 6.0 years old (SD = 3.7). Males exhibited an average individual inbreeding coefficient of 0.004 (SD = 0.014) and an average number of generations in captivity of 2.4 (SD = 0.9) (Table 1). The 7242 females were, on average, 6.6 years old (SD = 3.9). Females exhibited an average inbreeding coefficient of 0.004 (SD = 0.015) and an average number of generations in captivity of 2.4 (SD = 1) (Table 1). Detailed results of the statistical models can be found in the Supplementary Tables S2 and S3 online.

### Effects of age, inbreeding and number of generations in captivity

**Figure 1:**
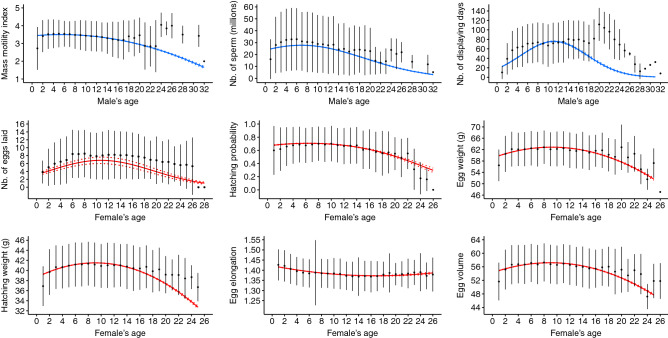
Predicted values of the reproductive parameters according to age. Solid blue lines represent male reproductive parameters while solid red lines represent female reproductive parameters. Dotted lines represent the 95% confidence intervals. Black filled points represent average phenotypic values computed on raw data and the vertical lines represent the associated standard deviations.

**Figure 2:**
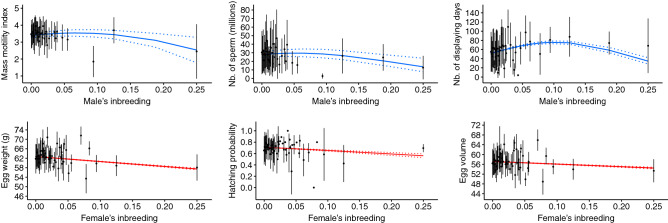
Predicted values of the reproductive parameters according to inbreeding. Solid blue lines represent male reproductive parameters while solid red lines represent female reproductive parameters. Dotted lines represent the 95% confidence intervals. Black filled points represent average phenotypic values computed on raw data and the vertical lines represent the associated standard deviations.

**Figure 3:**
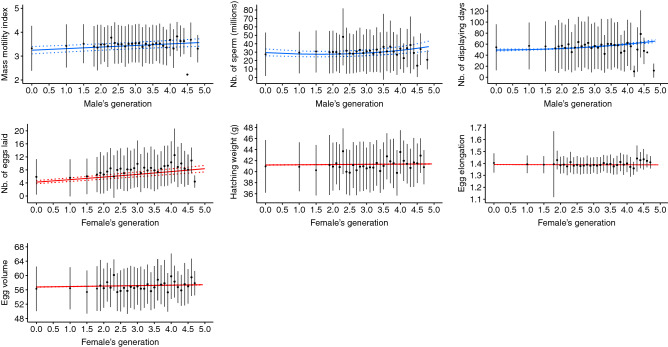
Predicted values of reproductive parameters according to number of generations in captivity. Solid blue lines represent male reproductive parameters while solid red lines represent female reproductive parameters. Dotted lines represent the 95% confidence intervals. Black filled points represent average phenotypic values computed on raw data and the vertical lines represent the associated standard deviations.

Strong effects of age were identified in both males and females (Table 3, Fig. 1). Substantial increases were observed in the number of sperm (+ 13%), number of displaying days (+ 237%) and number of eggs laid (+ 87%) before senescence occurred. Increases in other parameters were weak (≤ 5%; Table 4). Then, decreases in phenotypic values according to aging ranged from − 18% for hatching weight to − 99% for the number of displaying days (Table 4). Egg elongation slightly decreased (− 3%) before females reached 18 years of age and then slightly increased (+ 1%; Table 4). Note that since demographic variations with age may follow non-linear and relatively complex patterns^22^, the quadratic model does not necessarily reflect the precise shape of age-related variations. Additional figures of age-related variation based on less constrained models (generalized additive models) are presented in the Supplementary Figure S2 online. Increasing inbreeding was associated with substantial decreases in the mass motility index (− 28%), number of sperm (− 54%), number of displaying days (− 54%) and hatching probability (− 21%) (Fig. 2; Tables 3, 4). In other parameters, changes were weaker (≤ 8%; in egg weight and egg volume) or not statistically significant (i.e., in hatching weight, in the number of eggs laid and in egg elongation). Relationships between inbreeding F and males’ reproductive parameters were quadratic, and decreases started from F = 0.09 for the mass motility index, from F = 0.06 for the number of sperm and from F = 0.10 for the number of displaying days (Fig. 2). Larger confidence intervals with increasing F were mainly associated with the low number of highly inbred individuals. The proportion of individuals exhibiting an F equal to or greater than 0.1 ranged from 0.48 to 0.71% depending on the sampling associated with the parameter analyzed. Increasing number of generations in captivity was associated with increases in the mass motility index (+ 10%), number of sperm (+ 33%), number of displaying days (+ 33%) and number of eggs laid (+ 98%) (Tables 3; 4; Fig. 3). Changes were marginal in hatching weight (+ 0.5%), egg volume (+ 1%) and egg elongation (~ 0%), and not significant in either egg weight or hatching probability (Table 3). When comparing size effects (Table 4), decreases in reproductive parameters according to age were greater (i.e., larger percentage of variation after senescence onset) than changes according to inbreeding and number of generations in captivity except for the number of eggs laid, where the 85% decrease according to age was of the same magnitude as the 98% increase according to number of generations in captivity.

**Table 3:** Estimate values (i.e., slopes of the regression) and 95% confidence intervals of the effect of the three main explanatory variables (age, inbreeding and number of generations in captivity), their quadratic terms and interactions.

	Mass motility index	Nb. of sperm	Nb. of displaying days	Hatching weight	Egg weight	Egg volume	Hatching probability	Nb. of eggs laid	Egg elongation
Age	**− 0.037** [**− **0.072; **− **0.002]	0.0003 [**− **0.043; 0.043]	**0.469** [0.434; 0.504]	**0.462** [0.330; 0.594]	**0.369** [0.179; 0.558]	**0.428** [0.261; 0.595]	**− 0.105** [**− **0.133; **− **0.078]	**0.173** [0.146; 0.199]	**− 0.014** [**− **0.017; **− **0.011]
Age^2^	**− 0.043** [**− **0.044; **− **0.041]	**− 0.048** [**− **0.049; **− **0.046]	**− 0.171** [**− **0.177; **− **0.165]	**− 0.386** [**− **0.399; **− **0.372]	**− 0.542** [**− **0.563; **− **0.519]	**− 0.461** [**− **0.481; **− **0.442]	**− 0.063** [**− **0.071; **− **0.055]	**− 0.093** [**− **0.097; **− **0.088]	**0.002** [0.002; 0.003]
Inbreeding	**0.063** [0.014; 0.112]	0.032 [**− **0.004; 0.068]	**0.072** [0.030; 0.114]	**− **0.105 [**− **0.219; 0.009]	**− 0.271** [**− **0.455; **− **0.087]	**− 0.184** [**− **0.337; **− **0.030]	**− 0.048** [**− **0.075; **− **0.020]	**− **0.012 [**− **0.026; 0.004]	**− **0.00003 [**− **0.002; 0.002]
Inbreeding^2^	**− 0.008** [**− **0.013; **− **0.002]	**− 0.005** [**− **0.009; **− **0.001]	**− 0.006** [**− **0.011; **− **0.002]	n.s	n.s	n.s	n.s	n.s	n.s
Generation	**0.063** [0.032; 0.094]	**0.046** [0.023; 0.068]	**0.077** [0.053; 0.101]	**− 0.149** [**− **0.255; **− **0.043]	**− **0.167 [**− **0.336; 0.003]	**− 0.146** [**− **0.288; **− **0.004]	**− **0.014 [**− **0.041; 0.012]	**0.085** [0.070; 0.100]	**− 0.003** [**− **0.005; **− **0.002]
Generation^2^	n.s	**0.025** [0.010; 0.039]	**0.026** [0.007; 0.045]	n.s	n.s	n.s	n.s	n.s	n.s
Age + Inbreeding	0.001 [**− **0.000; 0.003]	n.s	n.s	n.s	**− 0.041** [**− **0.068; **− **0.015]	**− 0.036** [**− **0.060; **− **0.012]	n.s	n.s	**0.001** [0.000; 0.001]
Age + Generation	**0.008** [0.005; 0.011]	**− 0.005** [**− **0.007; **− **0.003]	**− 0.036** [**− **0.044; **− **0.028]	n.s	**− 0.066** [**− **0.102; **− **0.030]	n.s	n.s	**− 0.018** [**− **0.024; **− **0.011]	n.s
Inbreeding + Generation	n.s	n.s	**− 0.041** [**− **0.073; **− **0.008]	n.s.	n.s.	n.s.	n.s.	n.s.	**- 0.003** [− 0.005; − 0.000]

Estimates in bold indicate significance (*p* value < 0.05) while estimates of variables which effect was not significant and removed during model selection are not indicated (“n.s.”). More detailed results of models can be found in Supplementary Table S2, S3 online.

**Table 4:** Comparison of effect sizes through percentages of variation between the predicted value when the explanatory variable is at minimum (at age = 1; at F = 0; or at G = 0) and the predicted value when the explanatory variable is at maximum (at age = 32 for males and 25, 26, or 28 for females; at F = 0.25; or at G = 4.8 for males and 5.1 for females).

Reproductive parameters	Variation due to age		Variation due to inbreeding		Variation due to number of generations in captivity	
	Before inflection point (%)	After inflection point (%)	Before inflection point (%)	After inflection point (%)	Before inflection point (%)	After inflection point (%)
Mass motility index	+ 1	− 52	+ 4	− 28	+ 10	
Nb. of sperm	+ 13	− 88	+ 6	− 54	− 6	+ 33
Nb. of displaying days	+ 237	− 99	+ 47	− 54	+ 33	
Hatching weight	+ 5	− 18			+ 0.5	
Egg weight	+ 5	− 20	− 8			
Hatching probability	+ 4	− 57	− 21			
Nb. of eggs laid	+ 87	− 85			+ 98	
Egg elongation	− 3	+ 1			0	
Egg volume	+ 4	− 19	− 5		+ 1	

Two percentages are indicated when a quadratic relationship was included in the model. Percentage of variation was only computed when effect of a variable was significant (*p* value < 0.05).

### Interactions among age, inbreeding and number of generations in captivity

**Figure 4:**
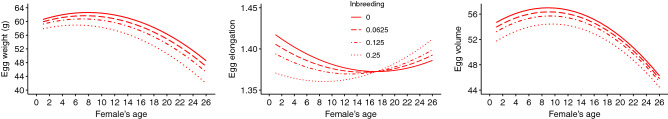
Predicted values of egg weight and egg elongation according to the interaction between age and inbreeding.

**Figure 5:**
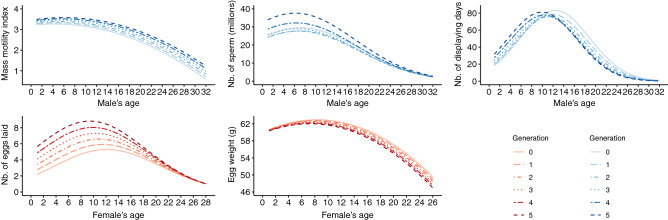
Predicted values of the reproductive parameters according to the interaction between age and number of generations in captivity.

**Figure 6:**
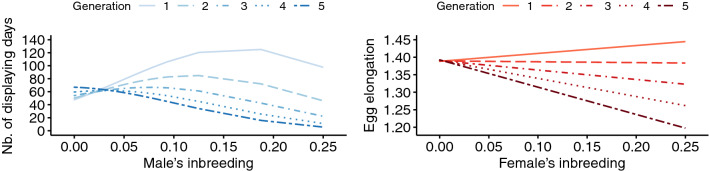
Predicted values of the number of displaying days and egg elongation according to the interaction between inbreeding and number of generations in captivity. The interaction between inbreeding and generation zero was not performed since founders (i.e., individuals of generation zero) were assumed to be neither inbred nor related.

The interaction between age and inbreeding had a significant effect (*p* value < 0.05) on the three parameters related to egg morphology (weight, elongation and volume; Table 3). The decrease in egg weight from aging females was steeper in highly inbred females, although the variation was weak (Fig. 4). Similarly, differences in the relationship between egg volume and female age essentially occurred in highly inbred females (Fig. 4). In egg elongation, an inflection point occurred at younger ages when the individual inbreeding coefficient increased (Fig. 4). In these three parameters, inbreeding induced an earlier senescent pattern. For other parameters, the interaction term was removed during model selection. The interaction between age and number of generations in captivity had a significant effect (*p* value < 0.05) on the mass motility index, number of sperm, number of displaying days, egg weight and number of eggs laid (Table 3). Similar patterns were obtained for the number of sperm, number of displaying days and number of eggs laid, with higher values at young and intermediate ages for higher numbers of generations in captivity and a convergence to similar lower values at old ages regardless of number of generations in captivity (Fig. 5). Such convergence was not observed for the mass motility index or egg weight. Regarding the interaction between inbreeding and number of generations in captivity, decreases in the number of displaying days and egg elongation according to inbreeding were greater in individuals issued from a higher number of generations in captivity (Table 3; Fig. 6).

## Discussion

Through a detailed study of nine reproductive parameters measured in 7242 females and 5053 males from a captive population of houbara, we showed that (1) age, inbreeding and number of generations in captivity had effects on reproductive parameters in a broadly consistent way across parameters; (2) the directions (signs) of these effects were consistent with some theoretical expectations, such as inbreeding depression or senescence theories; (3) effect sizes associated with age were substantially larger in our study system (approximately 5 generations and moderate levels of inbreeding) than those associated with inbreeding or number of generations in captivity; and (4) there were interactive effects of these factors on reproductive parameters.

The study of the effect of age revealed a substantial increase at young ages (< 12 years of age) in the number of sperm and number of displaying days for males and the number of eggs laid for females (Fig. 1; Table 4). This increase in reproductive performance at young ages is a phenomenon classically observed in animals and results from an increase in reproductive investment and/or the progressive appearance of reproductive phenotypes, e.g*.,* the appearance of courtship behavior^19^. For older ages and all parameters, the results were consistent with a pattern of senescence associated with a progressive decline in survival and/or fertility with individual aging^20,21^. In addition, senescence occurred very homogeneously between parameters (Fig. 1) and was associated with ample variations (Table 4) in both sexes. A remarkably similar senescence onset was observed for all parameters measured at approximately 8–12 years of age except for egg elongation (Fig. 1; Supplementary Figure S2 online). The diversity of parameters affected by senescence is consistent with studies in other vertebrate species, where senescence has been shown to affect sperm quality and quantity in males, such as in red wolf *Canis rupus*^6^ and *Pelvicachromis taeniatus*^23^; female juvenile production, such as in *Accipiter nisus*^65^ and great tit *Parus major*^66^; or the brood size of females, such as in great tit *Parus major*^66^. The results corroborate those of previous studies in houbara conducted in captivity at the phenotypic level^38–40^ and the genetic level^37^. In addition, a recent study of the reproductive parameters of captive-bred individuals released into the wild showed that the deleterious effects of senescence translated into free-ranging population reproductive success parameters, such as nest survival (i.e., the probability that at least one egg remains in the nest at a given time interval) or clutch size^36^. Although our study did not explore the physiological mechanisms underpinning the senescence patterns found in reproductive parameters, the results are consistent with previous studies that highlighted variations in endocrine components of reproduction with age as well as decreases in reproductive behavior and gonadal functions^67–69^. For instance, Ottinger et al.^69^ showed that the release of gonadotropin-releasing hormone-I decreased with aging in the Japanese quail *Coturnix japonica*. This hormone has a central role in the regulation of reproduction in both sexes, especially through the control of the secretion of FSH (hormone controlling spermatogenesis) and LH (hormone controlling ovulation). In contrast, Lecomte et al.^70^ found that reproductive performance deteriorated with age but the baseline physiology did not change in a wild population of wandering albatross *Diomedea exulans*. These studies suggest that reproductive senescence is based on a complex set of underlying mechanisms, especially at the behavioral and physiological levels.

The captive population of houbara studied here is characterized by a low level of inbreeding, i.e., the average F was 0.005 (SD = 0.017) and the proportion of individuals with an F greater than 0.1 ranged from 0.48 to 0.71%^32^. The results showed an overall negative effect of inbreeding on the studied reproductive parameters with large effect sizes, especially for male parameters and hatching probability (e.g., 54% reduction in the number of sperm per ejaculate when F increased from 0.06 to 0.25; Fig. 2; Table 4), despite the globally low level of inbreeding. Note that the low proportion of highly inbred individuals did not allow us to deeply investigate the magnitude and shape of inbreeding depression over a large range of inbreeding. However, the large dataset and the absolute number of individuals with F > 0.05 (N = 343) allowed us to assess the effects of inbreeding for moderate values of F and to compute robust predictions. Indeed, these results are consistent with previous studies^71^, especially in houbara, which found a negative effect of inbreeding on hatching success, growth, post hatching mortality and dispersal of captive-bred individuals released into the wild^33–35^. Interestingly, these overall reductions were non-linear in male reproductive parameters (Fig. 2). A non-linear relationship between inbreeding and phenotypic parameters has been reported previously in vertebrates^72^ and can be due to environmental variation (which was unlikely in our study considering the consistency and controlled conditions of the captive environment) or the involvement of epistasis in inbreeding depression^73^. As a result of directional epistasis, inbreeding depression is intensified for very high levels of inbreeding, although a linear relationship between inbreeding and inbreeding depression may be adequate for more moderate levels of inbreeding (i.e., for inbreeding below 0.2)^73^. In this context, the results highlighted an increased negative effect of inbreeding on reproductive parameters of males (i.e., the mass motility index, number of sperm per ejaculate and number of displaying days) when F reached values close to 0.10 (between 0.10 and 0.06 depending on the parameters; Fig. 2). This result reinforces the general recommendation accepted several decades ago urging conservation breeding programs to maintain average inbreeding levels in captive populations below 0.10^74^. Actually, this value is recognized as a threshold at which considerable negative effects of inbreeding on fitness may be observed^75^. Our study confirms the importance of accounting for inbreeding in the study of reproductive parameters. Inbreeding depression is a widely studied mechanism at the phenotypic level and it is of paramount importance in the conservation genetics of relict, captive or restored wild populations^8,76^. Because of its increasing severity in a stressful environment^77,78^, inbreeding depression has been shown to be more severe in the wild than in captivity^79^.

After age and inbreeding, the third factor impacting the reproductive parameters is the number of generations in captivity. Under certain conditions, captivity may induce phenotypic changes of plastic or genetic origin, as highlighted in previous studies^7,11,80^. Such changes might challenge the success of conservation breeding programs if they are associated with reduced fitness and a high risk of extinction of populations restored in the wild^9,12,81–83^. Here, we observed substantial effects of number of generations in captivity on some of the parameters studied. The present study was conducted at the phenotypic level and does not allow for a clear distinction between phenotypic plasticity and adaptation, which will require further investigation. In males, the increase in number of generations in captivity was associated with individuals exhibiting greater reproductive qualities (i.e., more motile sperm and in greater quantity; Fig. 3), which may be related to greater access to reproduction among males with high sperm production associated with artificial insemination^42^, even if the breeding protocol aims to equalize contributions through equalization of family size. Furthermore, the increase in the number of eggs laid per female with number of generations in captivity resulted from an increase in the number of clutches per breeding season. Complementary analyses (see Supplementary Results S1 online) highlighted that the reproductive period duration (i.e., number of days between the first and the last egg per breeding season) increased with number of generations in captivity, which was associated with a higher number of clutches; however, the number of eggs per clutch did not increase. On the other hand, changes in egg elongation and volume and hatching weight were weak (differences below 0.1 points for egg elongation and volume and below one gram for hatching weight) and associated with large confidence intervals (Fig. 3), suggesting a lack of biological meaning. Significant effects were found because of the large dataset. A potential explanation for the increases in reproductive performance is the expression of plasticity in response to captive conditions because reproductive performance is strongly connected to environmental conditions, especially food availability^84^. Thus, suboptimal conditions of captivity (e.g., veterinary care, ad libitum feeding and absence of predators) can shift the trade-off between survival and reproduction by relaxing constraints on survival, hence allowing for increased investment in reproduction (e.g., increases in the number of displaying days and in reproductive period duration). An additional argument toward the expression of plasticity in response to captive conditions is that houbara is a relatively long-lived species, with a maximum longevity of 15 years in the wild (32 years in captivity) and a generation time of 7.83 years^85^. Finally, the results highlighted phenotypic changes after only five generations in captivity (Table 4; Fig. 3). This finding is consistent with previous studies highlighting that phenotypic responses to a change in selection pressure can occur within a few generations in captivity^15,16^. Although these phenotypic changes may have large magnitudes, especially in the number of displaying days or number of eggs laid (Fig. 3), it is important to note that the average number of generations in captivity has stabilized since 2008 at approximately 2.5 (SD = 0.03) (see Supplementary Fig. S2 online) thanks to genetic management^32^. This allowed to minimize the risk of adaptation to captivity since it is dependent on the number of generations spent in captive conditions^7,14^. Although phenotypic changes observed across generations can be driven by phenotypic plasticity, they are also consistent with potential evolutionary changes and reinforce the need for suitable genetic management and appropriate breeding protocols to avoid adaptation to captivity, such as the regular addition of wild-bred individuals to the captive population. Nevertheless, whether the phenotypic changes observed over generations in captivity are plastic or adaptative, it is important to understand how these changes translate into the phenotype of released individuals and the dynamics and viability of the enhanced population, which are central to define the success of restoration programs^86^. A recent study^87^ in a reinforced houbara population highlighted that differences in reproductive performance between wild-bred and captive-bred released females were mainly driven by the period of release (i.e., females released in autumn performed better than those released in spring). This finding illustrates that translocation strategies represent a crucial component of conservation breeding program success beyond the management of captive populations.

Beyond the expected effects of age, inbreeding and number of generations in captivity, the present study allowed us to evaluate the relative importance and interactions of these factors. In terms of relative importance, the results showed that within ranges of variation encountered in the captive population of houbara (maximum number of generations of 5.1, relatively low inbreeding, age up to approximately 30 years), the effects of age (especially those associated with senescence) had a stronger magnitude than those of inbreeding or number of generations in captivity (Figs. 1, 2, 3, 4; Table 4). A comparison of the effect size may provide insights for the demographic and genetic management of conservation breeding programs. Because minimizing number of generations in captivity helps to prevent adaptation to captivity and can minimize the loss of genetic diversity^7^, one option is to increase generation length via reproduction among older individuals^2^. However, our results highlighted a predominant effect of aging compared to number of generations in captivity; in addition, recent results in houbara indicated an intergenerational link between aging and reproductive parameters^88^. Thus, managers must account for interactions between these factors and consider a trade-off between maximizing generation length and the age of the breeders. An interaction between inbreeding and age was found in three female parameters related to egg morphology. We found that greater senescence was associated with higher levels of inbreeding, which affected the egg weight and elongation; however, the effects of age and inbreeding were mostly additive, and the effect of age was strongest in the ranges of variation examined here (Fig. 4; Table 4). While there are several evolutionary theories explaining inbreeding depression and senescence, the dominant theories are based on the effects of spontaneous mutations with deleterious effects in both cases^27,28^, suggesting that inbreeding depression and senescence can interact. Many studies have empirically shown a positive interaction between the two phenomena, with greater inbreeding depression at older ages^6,23,25,26,28–30,89^, even if some studies did not find this relationship^90,91^.

The results also indicated a positive interaction between senescence and number of generations in captivity (Fig. 5). For the number of sperm per ejaculate, number of displaying days and number of eggs laid, changes observed over generations in captivity were essentially focused on young and intermediate ages, while the values of these parameters for the different number of generations in captivity converged at older ages. This finding can be attributed to a predominant effect of senescence at older ages compared to number of generations in captivity. A different pattern was obtained for the mass motility index and egg weight, for which differences between number of generations in captivity were greater at senescence. The selective forces are expected to vary with age, and this age-dependent variation in the magnitude (or even direction) of selection is the basis of evolutionary theories of senescence^21,27^. This hypothesis was highlighted empirically in relation to immunity^92^ and selection by predation^93^. The age-dependent selection suggested by the results is likely to be different from that in the natural environment for many reasons, such as the differences in selective pressures or captive genetic management, one principle of which is to extend the generation time by breeding older individuals.

Finally, although we did not formulate precise predictions on the interaction between inbreeding and number of generations in captivity, our results are consistent with the existence of such an interaction. In particular, the decrease in the number of displaying days according to inbreeding was stronger and started at a lower inbreeding coefficient for individuals with a higher number of generations in captivity (Fig. 6). This finding is consistent with an increase in the proportion of recessive or partially recessive segregating mutations in the population. Regarding egg elongation, variations in inbreeding effects according to number of generations in captivity raise the question of how egg shape impacts hatching success. Indeed, previous studies pointed out that changes in egg shape can be associated with variations in hatching success^46,47^, while in our case, hatching probability was not affected by the interaction between inbreeding and number of generations in captivity. Thus, while our results did not allow us to identify the mechanism responsible for the variation in the magnitude of inbreeding depression, they suggest increased negative effects of inbreeding over generations. Inbreeding depression can vary according to environmental harshness^94^ or purging^95^ or by increases in the proportion of segregating loci responsible for inbreeding depression with time, which is expected in the case of relaxation of selection or as a transitory effect of population size reduction.

## Conclusions

In captivity, reproductive performance is assumed to vary from one individual to another and over individuals’ lives, which is consistent with observations in the wild. The success of a conservation breeding program is related both to the long-term demographic functioning of the captive population and the potential contribution of released individuals to the growth of the free-ranging population. Therefore, it is essential to quantify the factors associated with demographic variations in captivity and determine whether these factors are related to individual life history, captive conditions, genetic dynamics of the captive population or interactions between these sources of variation. Here, we considered age, inbreeding level and number of generations in captivity, and studied their potential interactions and the relative magnitudes of their effects on reproductive parameters in a large captive population with up to 5 generations in captivity over a period of 24 years. Concerning age and inbreeding, our results are in keeping with the theoretical and empirical expectations related to aging theories and inbreeding depression but show that age has a larger size effect than inbreeding depression at these levels of inbreeding. Our results are also consistent with both hypotheses of plastic or genetic modifications related to captive conditions. At the scale of 5 generations in captivity, these intergenerational variations are on the same order of magnitude as inbreeding depression. In the context of genetic management of conservation breeding programs, our results confirm the importance of minimizing both inbreeding and number of generations in captivity to minimize the risk of inbreeding depression and adaptation to captivity, which, as highlighted here, can be mutually reinforcing. The prevalence of the effect of age on the studied parameters suggests that it is essential to take this factor into account in the management of both the in situ and ex situ components of conservation programs.

## Supplementary Information

Supplementary Informations.

Supplementary Tables.

## Data Availability

The data that support the findings of this study are available from the corresponding author upon reasonable request.

## Code availability

Code used to produce results are available from the corresponding author upon reasonable request.
