# Supplementary material for: Reproductive performance in houbara bustard is affected by the combined effects of age, inbreeding and number of generations in captivity
Source: Sci Rep. 2021 Apr 9;11:7813. doi: 10.1038/s41598-021-87436-z (PMC8035203; doi:10.1038/s41598-021-87436-z)
Supplement: Supplementary file 1 — Supplementary Informations. [file 41598_2021_87436_MOESM1_ESM.pdf]

Supplementary Information

Reproductive performance in houbara bustard is affected by the combined effects of age, inbreeding and number of generations in captivity

Robin Rabier[1,2,3]*, Loïc Lesobre[1,3], Alexandre Robert[2]

1 Reneco International Wildlife Consultant LLC, Abu Dhabi, United Arab Emirates

2 Centre d'Ecologie et des Sciences de la Conservation (CESCO), Muséum national d'Histoire naturelle, Centre National de la Recherche Scientifique, Sorbonne Université, CP 135, 57 rue Cuvier 75005 Paris, France

3 Emirates Center for Wildlife Propagation, Missour, Morocco

* Corresponding author: robin_rabier@hotmail.fr

Supplementary Table S1. Models used to analyze houbara reproductive parameters before model selection. The meaning of variables can be found in Methods section of the article.

	Parameters	Fixed-effect variables	Interactions	Random effect variables	Distribution function
**Males**	Mass motility index	Age, Age², Inbreeding, Inbreeding², Generation, Generation², Location, Origin, N_Sperm, Delay_Sperm, NbVisits	Age+Inbreeding<br>Age+Generation<br>Inbreeding+Generation	BirthYear<br>YearRecord<br>MaleID	Gaussian
	Number of sperm				Negative binomial
	Number of displaying days	Age, Age², Inbreeding, Inbreeding², Generation, Generation², Location, Origin			Negative binomial
**Females**	Number of eggs laid	Age, Age², Inbreeding, Inbreeding², Generation, Generation², Location, Origin, N_Egg, Delay_Egg	Age+Inbreeding<br>Age+Generation<br>Inbreeding+Generation	BirthYear<br>YearRecord<br>FemaleID	Zero-truncated negative binomial
	Hatching probability				Binomial
	Egg weight				Gaussian
	Hatching weight				Gaussian
	Egg elongation				Gaussian
	Egg volume				Gaussian

Supplementary Methods. Description of houbara sperm collection and characterization.

Sperm collections were performed using a dummy female to stimulate the male. Ejaculates were collected in a Petri dish positioned between the male and the dummy female and then analyzed in an adjacent laboratory. Contaminated ejaculates with feces or urates were discarded and the volume (± 1 µL) of undiluted ejaculate was measured using a microliter pipette and diluted with Lake 7.1 diluent (1:1). The mass motility index, a proxy of sperm quality and fertility potential in the species[1], was assessed under microscope (×100): (0) no motile sperm, (1) few motile sperm, (2) less than 50% of moderate motile sperm, (3) above 50% of motile sperm, (4) above 80% of highly motile sperm and (5) almost 100% of sperm showing rapid movement [2].

References

1. Vuarin, P. *et al.* Sperm competition accentuates selection on ejaculate attributes. *Biol. Lett.* **15**, 20180889 (2019).
2. Saint Jalme, M., Gaucher, P. & Paillat, P. Artificial insemination in Houbara bustards (Chlamydotis undulata): influence of the number of spermatozoa and insemination frequency on fertility and ability to hatch. *Reproduction* **100**, 93–103 (1994).

Supplementary Results S1. Complementary analysis of the reproductive period duration, the number of clutches per breeding season and clutch sizes in female houbara.

As a potential explanation of the increase in the number of eggs laid per breeding season with the number of generations, we investigated the reproductive period duration, the number of clutches per breeding season and clutch sizes. Reproductive period duration was computed as the number of days between the first and the last egg of the breeding season. Clutch sizes were computed as the number of eggs per each clutch of the breeding season. These three parameters were computed for each female using the same dataset as the one used to analyze the number of eggs laid. They were analyzed using the package glmmTMB 0.2.3[1] in R 3.6.1[2] with a Gaussian distribution function for the reproductive period duration and clutch sizes while a Poisson distribution function was used for the number of clutches. Models included age, the quadratic term of age, inbreeding and number of generations as fixed effect variable and the year of record, the birth year and female identity as factorial random effect variables. Results of models are presented below:

**Reproductive period duration**				
Variable	***Estimate***	***Std error***	***Z value***	***p-value***
(Intercept)	50.33	1.86	26.98	< 0.001
Age	10.48	0.55	19.11	< 0.001
Age^2	-3.72	0.10	-35.71	< 0.001
Inbreeding	-0.16	0.33	-0.47	0.64
Generation	3.08	0.32	9.49	< 0.001

**Number of clutches**				
Variable	***Estimate***	***Std error***	***Z value***	***p-value***
(Intercept)	1.48	0.03	41.29	< 0.001
Age	0.19	0.01	17.14	< 0.001
Age^2	-0.07	0.00	-31.80	< 0.001
Inbreeding	-0.00	0.01	-0.59	0.56
Generation	0.06	0.01	10.35	< 0.001

**Clutch sizes**				
Variable	***Estimate***	***Std error***	***Z value***	***p-value***
(Intercept)	1.75	0.02	82.44	< 0.001
Age	-0.02	0.01	-3.40	0.001
Age^2	-0.02	0.00	-12.68	< 0.001
Inbreeding	-0.01	0.00	-2.41	0.02
Generation	0.04	0.00	8.66	< 0.001

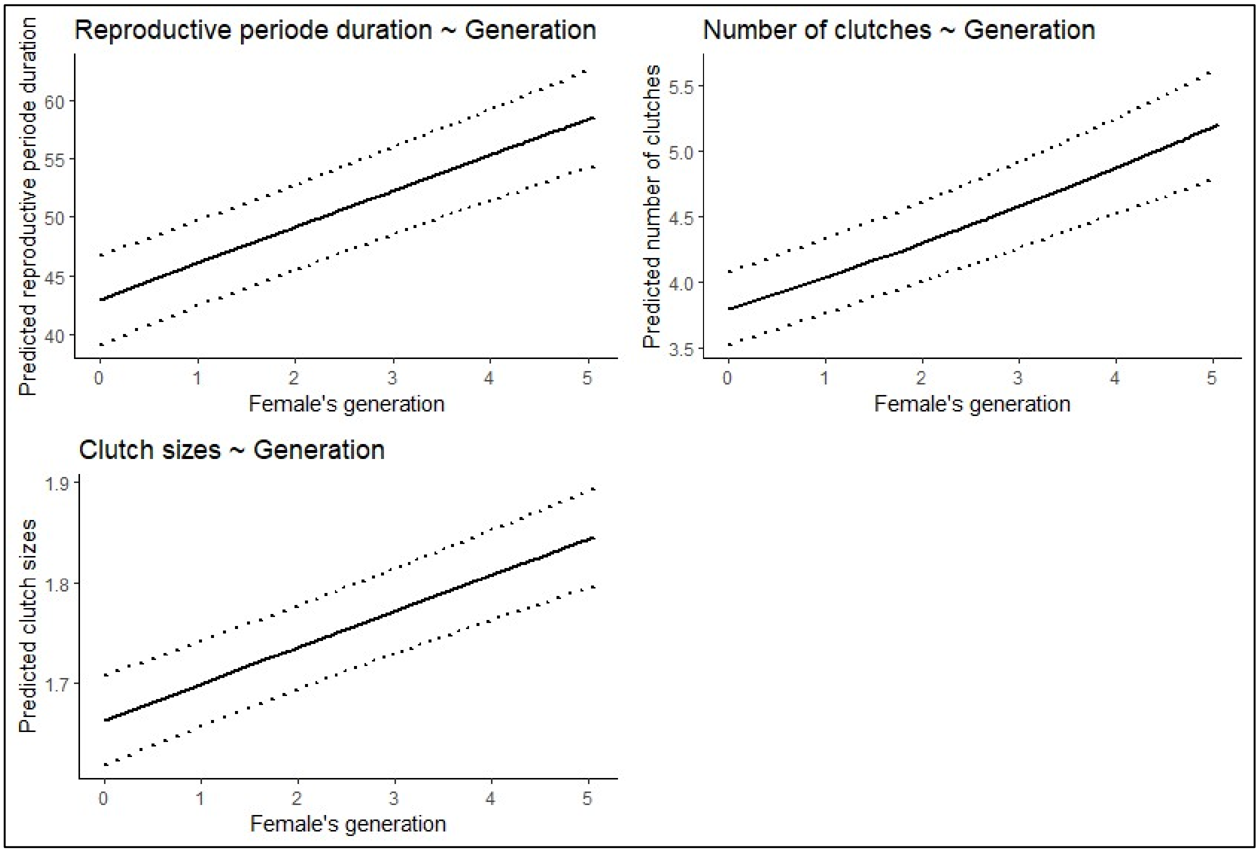

Figure 1. Predicted values of reproductive period duration, the number of clutches and clutch sizes of females houbara according to females' number of generations in captivity

Results showed significant increases in reproductive period duration, in the number of clutches and in clutch sizes according to number of generations in captivity. However, considering predicted values (Figure 1), the increase in clutch sizes was not biologically relevant because of its low magnitude (i.e., about 0.2 more eggs per laying after 5 generations). In addition, reproductive period duration and the number of clutches were respectively 91% [Pearson: cor=0.91] and 94% [Pearson: cor=0.94] correlated with the number of eggs laid while clutch sizes were only 51% [Pearson: cor=0.51] correlated with the number of eggs laid. Thus, the increase in the number of eggs laid was rather due to an increase in reproductive period duration associated with an increase in the number of clutches per breeding season than to an increase in clutch sizes.

References

1. Brooks, M. E. *et al.* glmmTMB Balances Speed and Flexibility Among Packages for Zero-inflated Generalized Linear Mixed Modeling. *The R Journal.* **9**, 378-400. (2017).

2. R Core Team. *R: a language and environment for statistical computing*. *R Foundation for Statistical Computing.* https://www.R-project.org. (2019).

Supplementary Figure S1. Temporal dynamic of the average number of generations of ECWP's captive population of houbara. Vertical bars indicate standard errors.

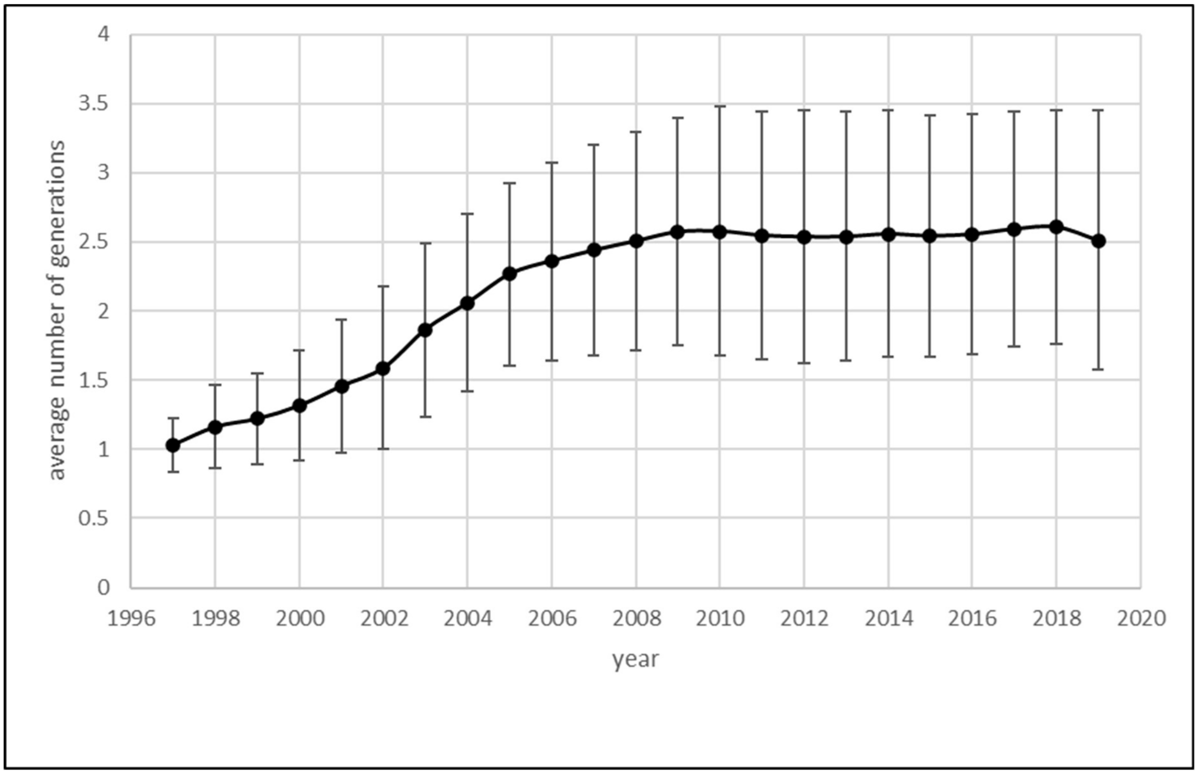

Supplementary Figure S2. Estimated smoothing curve (cubic regression spline) of Generalized Additive Mixed Effects Models (solid lines and dashed lines for 95% confidence intervals) of the reproductive parameters as functions of age within ECWP's captive population of houbara. X-axis represents age while y-axis represents smoothing component of age.

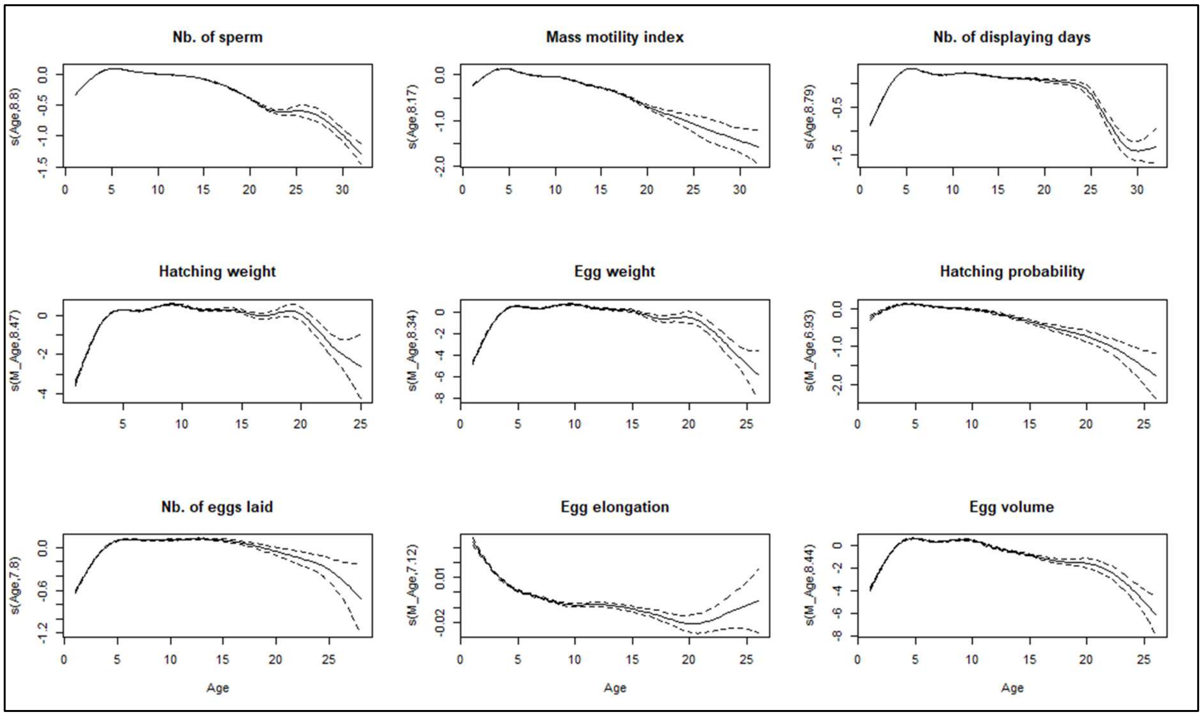

We used Generalized Additive Mixed Effects Models (GAMM) to assess the shape of the age effect on the nine reproductive parameters. GAMMs were fitted using the package mgcv 1.8-33[1] in R 3.6.1[2]. Individual identity was included as a random effect variable to account for pseudo-replication. Other random effects were not included because of convergence problems. GAMMs were fitted with Gaussian distribution functions for the mass motility index, hatching weight, egg weight, egg elongation and egg volume; with Poisson distribution functions for the number of sperm, number of displaying days and number of eggs laid; with a binomial distribution function for hatching probability. GAMMs allowed for more complex shapes than quadratic functions used in Generalized Linear Mixed-effects Models presented in the main results.

References

1. Wood S. N. *Generalized Additive Models: An Introduction with R*, 2nd edn. (Chapman and Hall/CRC, 2017). doi:10.1201/9781315370279

2. R Core Team. *R: a language and environment for statistical computing*. *R Foundation for Statistical Computing.* https://www.R-project.org. (2019).
